# Combining Serum Cystatin C with Total Bilirubin Improves Short-Term Mortality Prediction in Patients with HBV-Related Acute-On-Chronic Liver Failure

**DOI:** 10.1371/journal.pone.0116968

**Published:** 2015-01-28

**Authors:** Zhihong Wan, Yichen Wu, Jing Yi, Shaoli You, Hongling Liu, Zhiqiang Sun, Bing Zhu, Hong Zang, Chen Li, Fangfang Liu, Dongze Li, Yuanli Mao, Shaojie Xin

**Affiliations:** 1 Liver Failure Treatment and Research Center, Beijing 302 Hospital, Beijing, China; 2 Department of Health Statistics and Information Management, School of Public Health and Management, Chongqing Medical University, Chongqing, China; 3 Clinical Laboratory Center, Beijing 302 Hospital, Beijing, China; Duke University, UNITED STATES

## Abstract

**Background & Aims:**

HBV-related acute-on-chronic liver failure (HBV-ACLF) is a severe liver disease which results in a high mortality in China. To early predict the prognosis of the patients may prevent the complications and improve the survival. This study was aimed to develop a new prognostic index to estimate the survival related to HBV-ACLF.

**Methods:**

Consecutive patients with HBV-ACLF were included in a prospective observational study. Serum Cystatin C concentrations were measured by using the particle-enhanced immunonephelometry assay. All of the patients were followed for at least 3 months. Cox regression analysis was carried out to identify which factors were predictive of mortality. The area under the receiver operating characteristic curve (AUC) was used to evaluate the efficacy of the variates for early predicting mortality.

**Results:**

Seventy-two patients with HBV-ACLF were recruited between January 2012 and January 2013. Thirty patients died (41.7%) during 3-months followed up. Cox multivariate regression analysis identified serum cystatin C (CysC) and total bilirubin (TBil) were independent factors significantly (*P* < 0.01) associated with survival. Our results further showed that new prognostic index (PI) combining serum CysC with TBil was a good indicator for predicting the mortality of patients with HBV-ACLF. Specifically, the PI had a higher accuracy than the CTP, MELD, or MELD-Na scoring for early prediction short-term survival of HBV-ACLF patients with normal levels of serum creatinine (Cr). The survival rate in low risk group (PI < 3.91) was 94.3%, which was markedly higher than those in the high-risk group (PI ≥ 3.91) (17.4%, *P* < 0.001).

**Conclusion:**

We developed a new prognostic index combining serum CysC with TBil which early predicted the short-term mortality of HBV-ACLF patients.

## Introduction

Acute-on-chronic liver failure (ACLF) is a unique clinical entity that is characterized by acute onset, poor prognosis and high short-term mortality. It is defined as acute liver decomposition on the basis of chronic liver disease with mandatory jaundice, coagulopathy and recent development of complications [1, 2]. In China, HBV-related acute-on-chronic liver failure (HBV-ACLF) patients account for about 80% of ACLF cases because of a high incidence of chronic HBV infection. The lack of effective therapeutic methods for ACLF patients results in a poor prognosis of the disease in China [3, 4]. The further progress of ACLF may affect many organ functions. Kidney dysfunction is usually occurred in advanced liver diseases, which is related to a high mortality. Kidney function is commonly altered due to underlying circulatory abnormalities in patients with ACLF [5–7]. Early assessment and treatment of renal dysfunction of these patients may improve their prognosis.

Prognostic models are complex tools, which can be used to predict clinical outcomes. Many prognostic models which were used for liver cirrhosis have been used to evaluate the ACLF [8–10]. The model for end-stage liver disease (MELD) was commonly applied as a prognostic indicator in patients with ACLF. This score is formed from biochemical indicators: international normalized ratio (INR) for assessing coagulopathy, total bilirubin level for assessing liver dysfunction, and the serum creatinine (Cr) concentration as an indicator for assessing renal dysfunction. However, previous study has indicated that serum Cr may not be reliable for assessing the MELD scoring system [11]. Cr is an insensitive indicator for assessing acute renal dysfunction since it is highly dependent on extrarenal factors. Serum Cr levels may not change until ~50% of renal function has already been lost [12, 13]. Thus, much more reliable markers for estimation of renal function are required.

Serum cystatin C (CysC) is currently used in the prediction of acute kidney injury (AKI). Serum CysC concentration detects impairment of glomerular filtration rate (GFR) earlier than both Cr and endogenous creatinine clearance rate (Ccr) do [14, 15]. Previous studies have reported that serum CysC concentrations are more sensitive than Cr to predict hepatorenal syndrome in liver cirrhotic patients [16–18]. Our previous study has also shown that CysC levels may be an early biomarker to detect AKI development in HBV-ACLF patients who had normal serum levels of Cr [19].

In cirrhotic patients, serum CysC concentrations may also be a good marker for prognosis [17–18]. Our previous study has shown that CysC levels significantly correlated with MELD scoring system, suggesting that it should be a good marker for assessing the prognosis in these patients [19]. However, prognostic usefulness of serum CysC concentrations in HBV-ACLF patients has not been investigated previously. Thus, the study was aimed to evaluate whether serum CysC levels were associated with mortality, and further develop a new prognostic index to estimate the survival in patients with HBV-ACLF.

## Materials and Methods

### Patients

**Fig 1:**
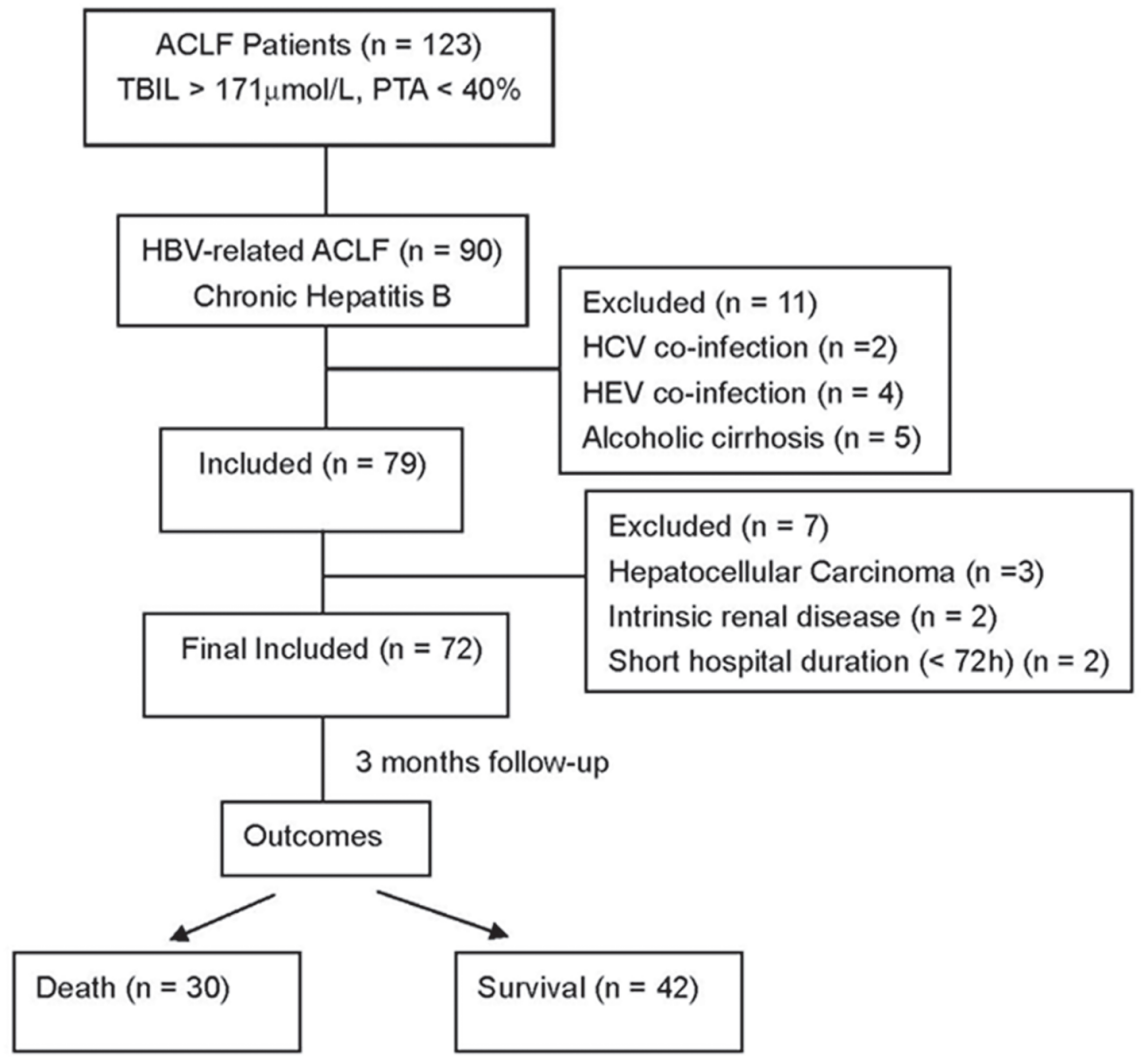
Study schema of enrolled patients and their outcomes.

The prospective observational study was conducted in Liver Failure Treatment and Research Center of Beijing 302 Hospital between January 2012 and January 2013. All of the patients with ACLF were recruited during this period. The Chinese criteria for diagnosis of ACLF were: (1) preexisting chronic liver diseases; (2) acute deterioration (usually within 4 weeks) with increasing jaundice (serum total bilirubin > 171.0 μmol/L or a daily elevation > 17.1 μmol/L); (3) plasma prothrombin activity (PTA < 40%) or international standard ratio (INR) ≥ 1.5 [2]. The criteria for ACLF have been widely used in China and are similar (but not exactly identical) with the criteria suggested by Asian Pacific Association for the Study of the Liver (APASL) [1]. Chronic HBV infection and liver cirrhosis were diagnosed according to the criteria which were suggested by the Chinese Society of Infectious Diseases, and the Chinese Society of Hepatology [20]. Fig. 1 showed the flow chart of the study group in our study. Total of 72 patients were finally enrolled and these patients were followed up for at least 3 months from the date when HBV-ACLF was diagnosed.

### Management of Patients

Every patient received comprehensive medical intervention including supportive therapy, intensive care monitoring, prevention and treatment for complications. Patients also received intravenous antibiotics, albumin, terlipressin, or proton pump inhibitors if required. Oral antiviral treatment including Lamivudine, Adefovir Dipivoxil, and Entecavir were applied to the patients in whom hepatitis B virus activated replication (HBV DNA ≥ 10^2^ copies/ml).

### Ethics Statement

The protocol was approved by the human Ethical Committee of Beijing 302 Hospital. All procedures were conformed to the ethical guidelines of the 1975 Declaration of Helsinki. Written informed consent was obtained from patients or guardians on behalf of the minors before entering the study protocol.

### Laboratory and clinical parameters

Serum samples from the patients were collected upon admission. All samples were stored at -80℃ until analysis. Biochemical tests were measured using routine biochemistry laboratory methods in Clinical Laboratory Center. Serum Cr levels were determined by using the modified Jaffe method (Beckman, Hamburg, Germany). Serum CysC concentrations were measured using the particle-enhanced immunonephelometry assay by the BN Prospec Nephelometer system (Dade Behring, Newark, DE, United States). MELD scoring system for HBV-ACLF was assessed as: *MELD* = 3.8*ln*(*TBil*) + 11.2*ln*(*INR*) + 9.6*ln*(*Cr*) + 6.4. MELD with incorporation of sodium (MELD-Na) scoring system was calculated as: MELD score + 1.59× (135-sodium). The Child-Turcotte-Pugh (CTP) scoring system was evaluated according to previous criteria [21].

### Statistical analysis

The results were presented as median (range) or numbers (percentage). Continuous variables were compared using Student’s t test. Mann-Whitney U test was used to compare the parameters with non-normal distribution. Categorical data were compared by the Chi-square test or Fisher’s exact test when appropriate. Cumulative incidences of mortality was estimated using the Kaplan–Meier method and was compared by log-rank test. Cox univariate and multivariate regression analysis was carried out to identify independent risk factors for predicting mortality. A new prognostic index (PI) was derived from Cox proportional hazards regression model. Receiver operating characteristic curves (ROCs) were performed to compare efficacy of MELD, CTP, MELD-Na scoring and PI for predicting the prognosis, using Medcalc 12.7.7 software. The areas under the receiver operating characteristic curve (AUCs) from different models were further compared by using DeLong’s method for ROC curve comparison. P < 0.05 (two tailed) was considered statistically significant. Data processing was performed with the SPSS statistical package for Windows (SPSS Inc, Chicago, United States).

## Results

### Baseline clinical characteristics of enrolled patients

From January 2012 through January 2013, 123 patients presenting as ACLF due to different etiological causes were screened. Seventy-two patients with HBV-ACLF were enrolled (Fig. 1). The basal clinical features of patients were provided in Table 1. These patients comprised 65 men (90.3%) and 7 women (9.7%), with a median age of 43 years (range 17–79 years). Forty-four (61.1%) of patients had liver cirrhosis. The median level of serum Na was 136 mmol/L (range 122 to 144 mmol/L). Hyponatremia (serum sodium level < 135 mmol/L) was documented in 31 patients (43.1%). The median CysC concentration was 1.52 mg/L (range 0.81 to 3.91 mg/L). Serum CysC concentration was above normal value (1.16 mg/L) in 83.3% of patients (60/72). Most of the patients (80.6%, 58 patients) had a normal serum level of Cr. The median MELD, MELD-Na, and CTP scoring were 25 (range 18 to 42), 26 (range 4 to 48), and 11 (range 9 to 13), respectively. There were 5 patients (6.9%) diagnosed as acute kidney injury at admission. The most common complications were ascites, spontaneous bacterial peritonitis (SBP) and hepatic encephalopathy (HE), which comprised 77.8%, 19.4% and 16.7% of patients, respectively. Of note, only grade I or II HE presented in these patients.

**Table 1:** Clinical Characteristics of enrolled patients.

Parameters	All patients (n = 72)
Age (yr)	43 (17–79)
Male, n (%)	65 (90.3%)
Alanine aminotransferase (IU/L)	107 (18–2495)
Albumin (g/L)	29 (19–45)
Blood Urea Nitrogen (mmol/L)	5.1 (1.9–15.5)
Sodium (mmol/L)	136 (122–144)
Total bilirubin (mg/dL)	18.9 (10.1–42.6)
Creatinine (mg/dL)	1.0 (0.7–3.7)
International normalized ratio (INR)	1.9 (1.5–3.4)
White Blood Cells (10^9^/L)	6.7 (1.7–17.5)
HBV DNA (Log10 copies/ml)	4.4 (1.6–9.5)
Cirrhosis, n (%)	44 (61.1%)
MELD scoring	25 (18–42)
MELD-Na scoring	26 (4–48)
CTP scoring	11 (9–13)
Cystatin C (mg/L)	1.52 (0.81–3.91)
Ascites, n (%)	56 (77.8%)
Spontaneous bacterial peritonitis, n (%)	14 (19.4%)
Hepatic encephalopathy, n (%)	12 (16.7%)
Acute kidney injury, n (%)	5 (6.9%)
Antiviral Therapy, n (%)	70 (97.2%)

All data were present as median (min-max) or number (%).

MELD: The model for end-stage liver disease

MELD-Na: MELD with incorporation of sodium

CTP: Child-Turcotte-Pugh

Antiviral therapy was applied to 70 patients (97.5%). The next of kin of two patients refused to receive antiviral therapy due to disease severity. As shown in Table 2, thirty patients (42.9%) received antiviral therapy before enrollment, of which 15 received Lamivudine and/or Adefovir Dipivoxil, 15 received Entecavir, respectively. The antiviral therapy was continuing applied to the patients after enrollment. Forty patients (57.1%) were given Entecavir within 72 hours after enrollment. There were no adverse events in these patients.

**Table 2:** Antiviral therapy for the patients.

Antiviral therapy (n = 70) ^a^	Before enrollment	After enrollment ^b^
Lamivudine	6 (8.6%)	/
Adefovir Dipivoxil	1 (1.4%)	/
Lamivudine plus Adefovir Dipivoxil	8 (11.4%)	/
Entecavir	15 (21.4%)	40 (57.1%)
Total	30 (42.9%)	40 (57.1%)

Data were present as number (%)

a Two patients refused to receive antiviral therapy.

b Antiviral therapy was applied in patients within 72 hours after enrollment.

### Followed up of Patients

**Fig 2:**
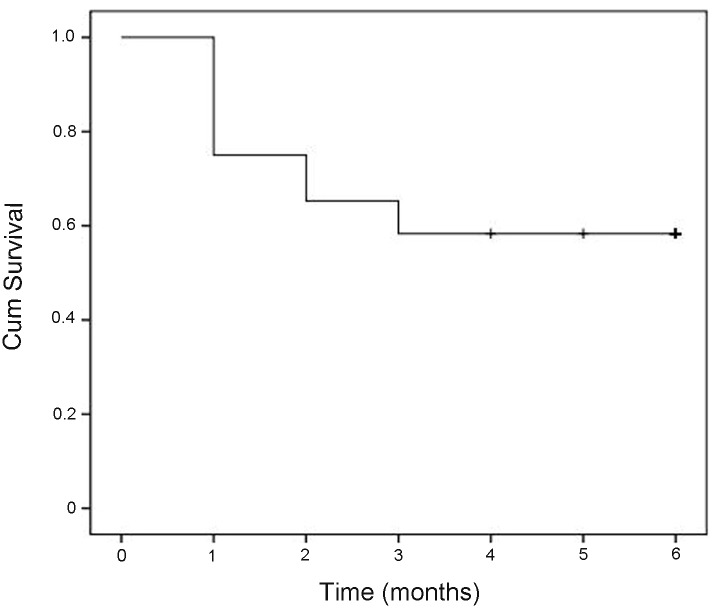
Kaplan–Meier curve showing mortality in patients with HBV-related acute-on-chronic liver failure.

All of the patients were followed-up for at least 3 months after enrollment. None of these patients accepted liver transplantation. Kaplan-Meier survival curve was shown in Fig. 2. Thirty patients (41.7%) died during follow-up period. The main causes of death were multiple organ failure (30.0%), septic shock (23.3%), hypovolemic shock (16.7%), cerebral edema/cerebral hernia (10.0%), and myocardial infarction (3.3%). Five patients (16.7%) died with unknown causes (Table 3). During this period, the main complications were bacterial infection, intractable ascites, hepatic encephalopathy, acute kidney injury and gastrointestinal hemorrhage. The frequencies of complications development were significant higher in non-survivors than those in survivors (all *P* < 0.05, Table 4).

**Table 3:** Main causes of death at 3 months after study enrollment.

Causes of death	Patients (n = 30)
Multiple organ failure	9 (30.0%)
Septic shock	7 (23.3%)
Hypovolemic shock	5 (16.7%)
Cerebral edema/Cerebral hernia	3 (10.0%)
Myocardial infarction	1 (3.3%)
Causes unknown	5 (16.7%)

Data were present as number (%)

**Table 4:** Main complications in enrolled patients during 3-months follow up.

Main complications	Survivors (n = 42)	Non-survivors (n = 30)	P value
Hepatic encephalopathy	6 (14.3%)	14 (46.7%)	0.002
Acute kidney injury	3 (7.1%)	11 (36.7%)	0.002
Gastrointestinal hemorrhage	2 (4.8%)	6 (20%)	0.043
Intractable ascites	3 (7.1%)	17 (56.7%)	<0.001
Bacterial infection	13 (30.9%)	20 (66.7%)	0.014

Data were present as number (%)

### Baseline Serum CysC concentrations and prognosis in patients with HBV-ACLF

The increase of CysC levels was significantly related to MELD score in our previous study; we next investigated the association between CysC concentrations and mortality in patients with HBV-ACLF.

As shown in Table 5, the baseline clinical and laboratory features of survivors and non-survivors were summarized. Cox univariate analysis showed that age, BUN, INR, TBil, CysC, MELD and MELD-Na scoring were significantly associated with mortality. Serum levels of CysC (β, 0.933; RR, 2.54; 95% CI, 1.59–4.08; *P* = 0.001) and TBil (β, 0.075; RR, 1.08; 95% CI, 1.03–1.12; *P* = 0.001) were independent predictive factors for the prognosis in multivariate analysis. Prognostic index (PI) for 3-month mortality was created as follow: *PI* = 0.933 × *CysC*(*mg*/*L*) + 0.075 × *TBil*(*mg*/*dL*).

**Table 5:** Comparison of baseline clinical characteristics between survivors and non-survivors.

Parameters	survivor (*n* = 42)	Non-survivor (*n* = 30)	*P* value (Cox univariate)	*P* value (Cox multivariate)	RR (95% CI)
Age (yr)	41.5 (17–60)	44.5 (22–79)	0.019		
Male, n (%)	40 (95.2%)	25 (83.3%)	0.389		
Alanine aminotransferase (IU/L)	169 (21–1499)	94 (18–2495)	0.187		
Albumin (g/L)	29.5 (19–45)	29 (23–41)	0.803		
Blood Urea Nitrogen (mmol/L)	4.2 (1.9–12.1)	6.4 (2.2–15.5)	0.039		
Sodium (mmol/L)	136 (126–144)	134 (122–143)	0.091		
Total bilirubin (mg/dL)	17.3 (10.1–30.9)	24.7 (12.8–42.6)	< 0.001	0.001	1.08(1.03–1.12)
Creatinine (mg/dL)	1.0 (0.7–1.8)	1.1 (0.7–3.7)	0.105		
International normalized ratio (INR)	1.8 (1.5–2.7)	2.0 (1.5–3.4)	0.015		
White Blood Cells (10^9^/L)	6.3 (1.7–15.4)	7.1 (2.5–17.5)	0.057		
HBV DNA (Log10 copies/ml)	4.8 (1.6–8.1)	3.2 (1.6–9.5)	0.224		
Cirrhosis, n (%)	25 (59.5%)	19 (63.3%)	0.744		
CTP scoring	11 (9–13)	11.5 (9–13)	0.062		
MELD scoring	24 (18–30)	27 (21–42)	0.001		
MELD-Na scoring	24 (4–37)	33 (14–48)	< 0.001		
Cystatin C (mg/L)	1.36 (0.81–2.91)	1.92 (1.21–91)	< 0.001	0.001	2.54(1.59–4.08)
Ascites, n (%)	32 (76.2%)	24 (80%)	0.701		
Spontaneous bacterial peritonitis, n (%)	8 (19.1%)	6 (20%)	0.920		
Hepatic encephalopathy (I–II), n (%)	6 (14.3%)	6 (20%)	0.521		
Acute kidney injury, n (%) *	1 (2.4%)	4 (13.3%)	0.153		
Antiviral therapy, n (%)	42 (100%)	28 (93%)	0.090		

All data were present as median (min-max) or number (%).

* Fisher’s Exact Test

**Fig 3:**
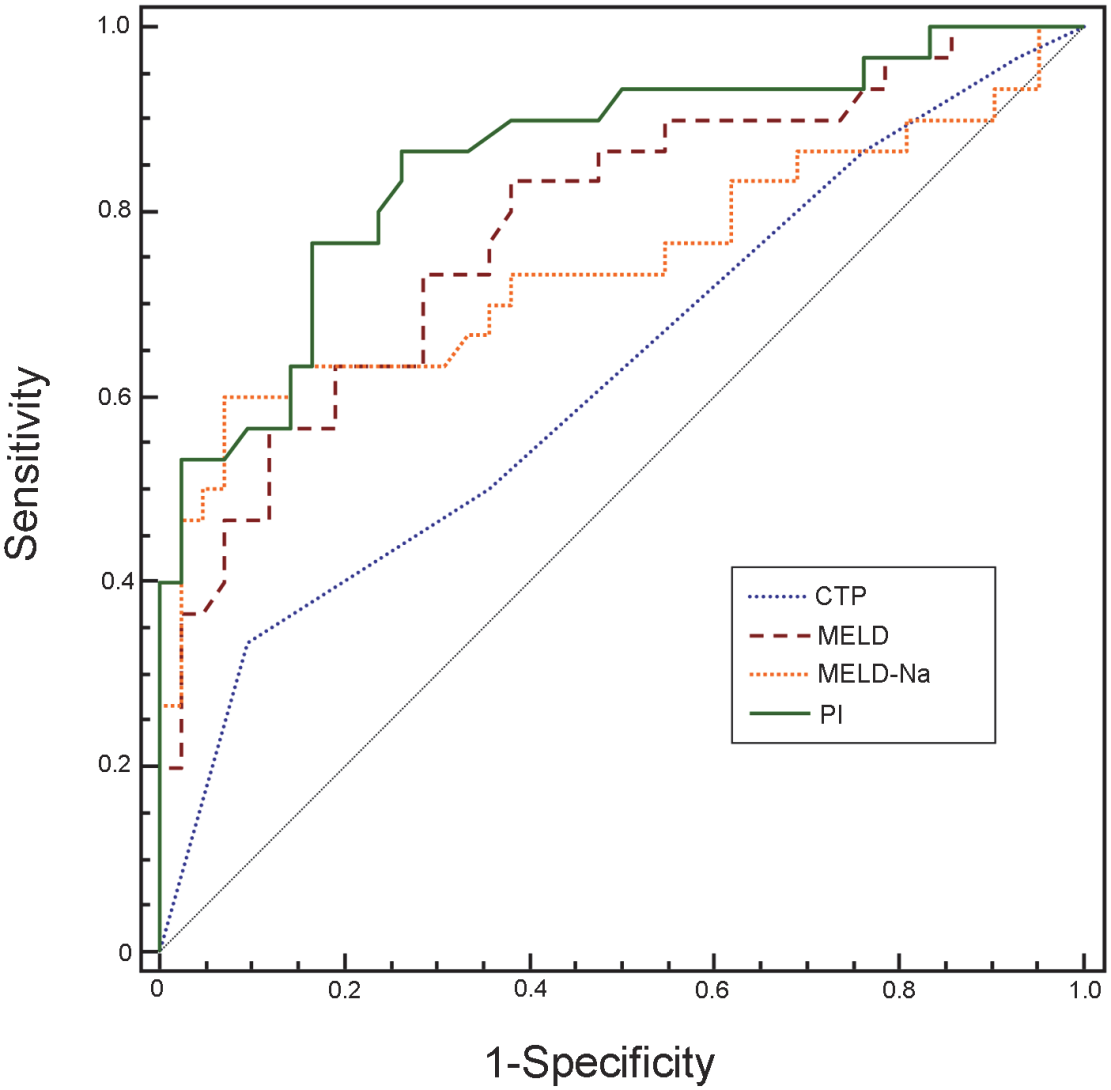
Receiver operating characteristic curves (ROC) for evaluating 3-months mortality in patients with HBV-related acute-on-chronic liver failure. The area under ROC curve (95% CI) of the new PI, CTP, MELD and MELD-Na scoring systems were 0.86 (0.75–0.93), 0.63 (0.50–0.74), 0.79 (0.67–0.87) and 0.74 (0.63–0.84), respectively. MELD: The model for end-stage liver disease. MELD-Na: MELD with incorporation of sodium. CTP: Child-Turcotte-Pugh. PI: prognostic index

Then ROC analysis was carried out to compare the efficacy of CTP, MELD, MELD-Na scoring, and PI in predicting 3-months mortality (Fig. 3). AUC for PI was 0.86, which was significantly larger than that for CTP scoring (0.63, *P* < 0.01). There were no significant differences between PI and MELD (0.79) or MELD-Na scoring (0.74) for prediction of mortality in these patients (*P* > 0.05, DeLong’s method for ROC curve comparison).

### Prediction value of serum CysC in HBV-ACLF patients with normal levels of serum Cr

Most of the patients had normal Cr levels in our study, which may make MELD scoring system less powerful in predicting prognosis for these patients. We next sought to examine whether serum CysC concentrations were related to mortality in HBV-ACLF patients who had normal serum levels of Cr.

**Fig 4:**
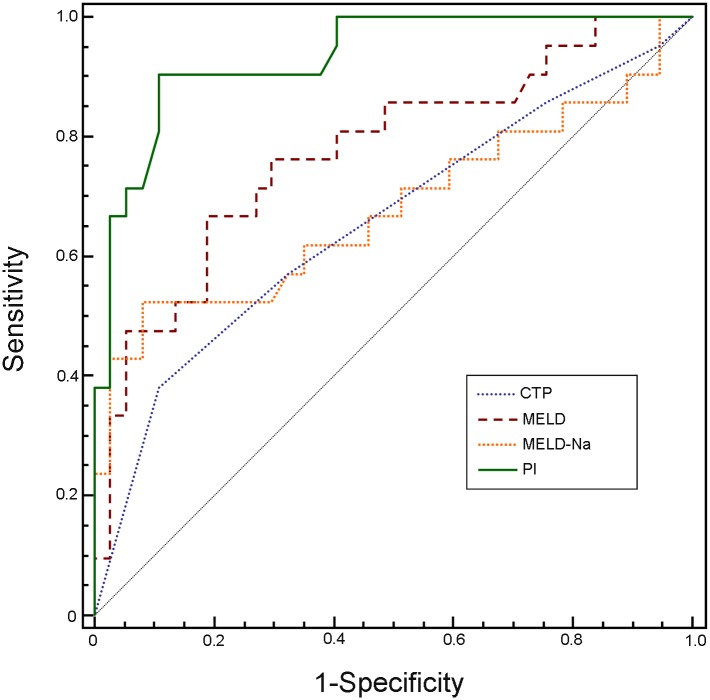
Receiver operating characteristic curves (ROC) for evaluating 3-month mortality in patients of HBV-related acute-on-chronic liver failure with normal levels of serum creatinine. The area under ROC curve (95% CI) of the new PI, CTP, MELD and MELD-Na scoring systems were 0.93 (0.84–0.98), 0.66 (0.52–0.78), 0.77 (0.64–0.87) and 0.68 (0.54–0.80), respectively. MELD: The model for end-stage liver disease. MELD-Na: MELD with incorporation of sodium. CTP: Child-Turcotte-Pugh. PI: prognostic index

**Fig 5:**
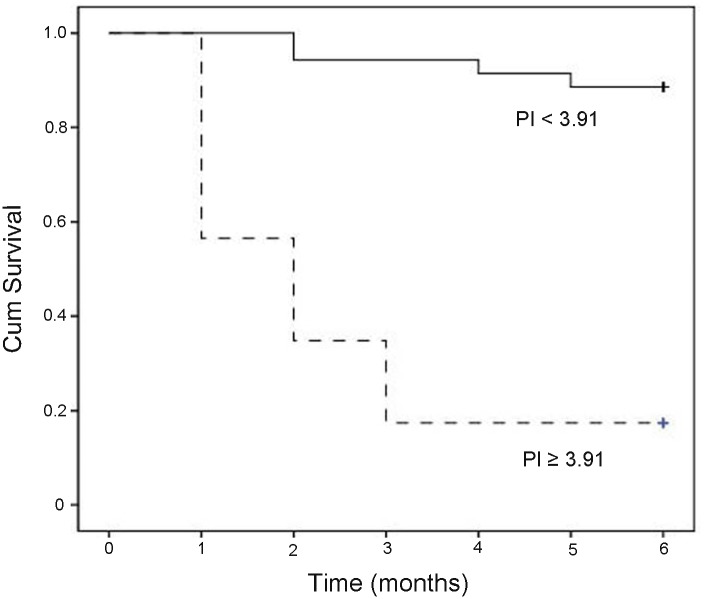
Survival curve in relation to prognostic index (PI) at admission in patients of HBV-related acute-on-chronic liver failure with normal levels of serum Cr. Dashed line represented the high-risk group (PI ≥ 3.91) and plain line represented the low-risk group (PI < 3.91). The survival rate in low risk group (PI < 3.91) was 94.3%, which was markedly higher than those in the high-risk group (PI ≥ 3.91) (17.4%, *P* < 0.001).

There were 58 patients with normal serum levels of Cr. During 3-months follow-up, there were 6 patients who had high CysC and normal creatinine levels at inclusion developed AKI. The average serum CysC concentration in these patients was 1.99±0.40 mg/L, which was significant associated with AKI development (data not shown, similar to our previous study, ref.19). Thirty-seven patients survived and 21 died. Approximate 95% (20/21) of non-survivors had increased serum levels of CysC, with median levels of 1.78 mg/L (range: 1.16–3.04 mg/L). On univariate analysis (Table 6), the prognostic factors with mortality were: CysC, INR, TBil, CTP, MELD and MELD-Na scoring (*P* < 0.05). The indicators (age, INR, TBil, CysC, CTP, MELD and MELD-Na scoring) which had *P* < 0.1 were entered to Cox multivariate analysis. The results revealed that serum levels of CysC (β, 1.593; RR, 4.92; 95% CI, 1.96–12.35; *P* = 0.006) and TBil (β, 0.068; RR, 1.07; 95% CI, 1.02–1.12; *P* = 0.01) were independent indicators to predict prognosis in the patients (Table 6). PI for 3-month mortality was created as follow: *PI* = 1.593 × *CysC*(*mg*/*L*) + 0.068 × *TBil*(*mg*/*dL*). The efficacy of CTP, MELD, MELD-Na scoring, and PI in predicting 3-month mortality was compared by ROC analysis (Fig. 4). As shown in Table 7, the AUCs for CTP, MELD, MELD-Na and PI were 0.66 (*P* = 0.041), 0.77 (*P* = 0.0001), 0.68 (*P* = 0.031), and 0.93 (*P* < 0.0001), respectively. Furthermore, the AUC for PI was markedly larger than those for CTP (z = 3.178, *P* = 0.001), MELD (z = 2.488, *P* = 0.011) and MELD-Na scoring (z = 2.256, *P* = 0.003), suggesting PI had a greater accuracy for early prediction 3-months mortality of these patients. Sensitivity and specificity for predicting 3-months mortality were 91% and 89%, respectively, with a threshold value of 3.91. Survival curve in relation to prognostic index (PI) at admission in patients of HBV-ACLF with normal levels of serum Cr was shown in Fig. 5. The survival rate in low risk group (PI < 3.91) was 94.3%, which was markedly higher than those in the high-risk group (PI ≥ 3.91) (17.4%, *P* < 0.001).

**Table 6:** Comparison of baseline clinical characteristics between survivors and non-survivors who had a normal serum Cr.

Parameters	survivor (*n* = 37)	Non-survivor (*n* = 21)	*P* value (Cox univariate)	*P* value (Cox multivariate)	RR (95% CI)
Age (yr)	42 (17–60)	43 (34–75)	0.059		
Male, n (%)	36 (97.3%)	18 (85.7%)	0.094		
Alanine aminotransferase (IU/L)	149 (25–1499)	102 (18–2495)	0.430		
Albumin (g/L)	30 (19–45)	28 (23–38)	0.473		
Blood Urea Nitrogen (mmol/L)	3.9 (1.9–7.3)	4.0 (2.2–8.1)	0.497		
Sodium (mmol/L)	136 (126–144)	133 (122–143)	0.184		
Total bilirubin (mg/ml)	16.7 (10.1–30.9)	24.4 (12.9–41.8)	<0.001	0.01	1.07 (1.02–1.12)
Creatinine (mg/ml)	1.0 (0.7–1.2)	1.0 (0.7–1.2)	0.779		
International normalized ratio (INR)	1.8 (1.5–2.7)	2.1 (1.5–3.8)	0.010		
White Blood Cells (10^9^/L)	6.1 (1.7–15.4)	6.8 (2.6–17.5)	0.219		
HBV DNA (Log10 copies/ml)	4.9 (1.8–8.1)	2.9 (1.6–9.5)	0.169		
Cirrhosis, n (%)	23 (62.2%)	13 (61.9%)	0.732		
CTP scoring	11 (9–13)	12 (9–13)	0.041		
MELD scoring	23 (18–30)	27 (21–37)	< 0.001		
MELD-Na scoring	24 (4–37)	31 (14–48)	< 0.001		
Cystatin C (mg/L)	1.34 (0.81–1.86)	1.78 (1.16–3.04)	< 0.001	0.006	4.92 (1.96–12.35)
Ascites, n (%)	28 (75.7%)	16 (76.2%)	0.965		
Spontaneous bacterial peritonitis, n (%)	7 (18.9%)	5 (23.8%)	0.659		
Hepatic encephalopathy (I–II), n (%)	5 (13.5%)	5 (23.8%)	0.318		

All data were present as median (min-max) or number (%).

**Table 7:** AUCs for ROC and cutoff value for predicting prognosis of HBV-ACLF patients with normal levels of serum Cr.

Parameters	Cutoff value	AUC (95% CI)	Sensitivity (%)	Specificity (%)	P value
MELD scoring	25.4	0.77 (0.64–0.87)	67	81	0.0001
MELD-Na scoring	30.6	0.68 (0.54–0.80)	52	92	0.031
CTP scoring	12	0.66 (0.52–0.78)	38	89	0.041
PI	3.91	0.93 (0.84–0.98)	91	89	<0.0001

MELD: The model for end-stage liver disease

MELD-Na: MELD with incorporation of sodium

CTP: Child-Turcotte-Pugh

PI: Prognostic index

## Discussion

HBV-ACLF accounts for most of the ACLF cases in China. Lacking effective therapeutic methods causes a high mortality rate in these patients. Thus, early predict the prognosis of these patients may reduce the complications and improve the survival.

The prognostic models evaluated for survival of ACLF patients used previously were CTP, MELD, MELD-Na scoring systems, Sequential Organ Failure Assessment (SOFA), chronic liver failure [CLIF]-SOFA score, and acute physiology and chronic health evaluation scoring system (APACHE-II) [7, 10, 22–24]. As is well known, CTP, MELD and MELD-Na scoring systems are used mainly in patients with decompensated cirrhosis. HBV-ACLF is a severe situation occurred under existing chronic liver disease with or without cirrhosis, which represents a complex situation which is different from cirrhosis [1, 6]. These models may not suitable to determine survival among patients with HBV-ACLF in our study, which was about 40% of patients without liver cirrhosis. Meanwhile, SOFA, CLIF-SOFA and APACHE-II scoring systems were not performed because the indicators, such as Glasgow Coma Scale scores or partial pressure of oxygen, and arterial/fraction of inspired oxygen, were difficulty to obtain since the patients were not in the ICU department in this study.

Patients with ACLF have immunological defects that are similar to those with sepsis. These patients may further progress to shock or multiple organ failure [25]. The studies from the Asian Pacific Association for the Study of the Liver (APASL) and the European Association for the Study of the Liver (EASL) have reported that kidney failure was a risk indicator to early predict mortality in ACLF patients [1, 6]. The renal failure in presence of liver failure may be progressed insidious or rapid. Serum Cr is a widely used factor for assessing renal function, while it is insensitive and may not change until renal function is severely impaired [26]. Recently, several studies have reported that CysC is more early and accurate than Cr for assessing kidney dysfunction and prognosis in cirrhotic patients [16–17, 27]. The data concerning CysC levels in early prediction for the outcomes of patients with HBV-ACLF are unavailable.

There were 72 patients with HBV-ACLF were enrolled in our study. Among them, serum CysC concentration was above normal value (1.16 mg/L) in 83.3% of patients, suggesting that mild renal dysfunction may occur. About 42% of patients died during 3-month follow-up period, indicating that HBV-ACLF is till a serious healthy problem in China. Cox regression analysis showed that independent factors which were related to survival were serum CysC (OR: 2.54), and Tbil (OR: 1.08). The novel prognostic index combining serum CysC with TBil was better than CTP scoring to predict 3-months mortality in HBV-ACLF patients, which in coordinate with MELD and MELD-Na scoring systems. The CTP scoring was less powerful for prediction outcome of patients with ACLF than MELD scoring as similar as reported before [22, 28].

Considering most of the patients in our study had normal levels of Cr, we further investigated prediction indicators in these patients. Cox multivariate regression analyses showed that serum levels of CysC (OR: 4.92) and TBil (OR: 1.07) were independent indicators to predict the prognosis. The forecast accuracy of new prognostic index was 93%. By comparing the AUCs, we revealed that the prognostic index had a higher predict value than the CTP, MELD and MELD-Na scoring systems (all *P* < 0.01). The new prognostic index outnumbered CTP scoring by 27% (from 0.66 to 0.93); MELD scoring by 16% (from 0.77 to 0.93); and MELD-Na scoring by 25% (from 0.68 to 0.93). Survival was significantly lower in patients with prognostic index above 3.91. These results indicated that combining serum CysC and TBil to improve accuracy in predicting short-term survival of patients with HBV-ACLF who had normal levels of serum Cr.

Our findings suggested that MELD scoring system was less efficient than PI to predict the prognosis of HBV-ACLF patients who had normal levels of serum Cr, which were inconsistent with previous studies [24, 28–29]. The main point of the differences is that serum CysC instead of Cr was used in assessing renal function in our study. About 96% of non-survivors in patients with normal Cr levels had increased serum CysC, indicating renal dysfunction already present in these patients. Thus, the prediction value of MELD scoring system for prognosis is obviously insufficient. Present complications such as hepatorenal syndrome and hepatic encephalopathy were identified as independent prognostic factors for mortality in ACLF patients previously [28, 30]. However, the incidences of the complications not differed between survivors and non-survivors in our study. The difference was mainly owing to the different severity of the disease between the cohorts. Compared with patients in the previous study who presented 20–40% HRS or 30–40% HE, the patients in our study were in early stage of ACLF with less disease severity. Thus, the new prognostic index combining serum CysC and Tbil was more accurate to early predict prognosis for HBV-ACLF patients.

To sum up, serum CysC and TBil concentrations were independently related to the poor prognosis in patients with HBV-ACLF. Our results showed that new prognostic index combining serum CysC with TBil was a good indicator for early predicting the short-term mortality in patients with HBV-ACLF. Specifically, the PI had a higher accuracy than the CTP, MELD, or MELD-Na scoring systems to early predict short-term mortality of HBV-ACLF patients who had normal serum levels of Cr. Unfortunately, the results wasn’t tested on external cohorts since serum CysC isn’t routinely measure in ACLF patients. Additional multicentre studies needed to validate the clinical usefulness of the new prognostic index.
